# Supplementary material for: DMRforPairs: identifying Differentially Methylated Regions between unique samples using array based methylation profiles
Source: BMC Bioinformatics. 2014 May 15;15:141. doi: 10.1186/1471-2105-15-141 (PMC4046028; doi:10.1186/1471-2105-15-141)
Supplement: Additional file 2 — DMRforPairs output for the comparison of A431-MCF7 and NA17018-NA17105. Please start from the HTML files in each folder. Available via the BMC Bioinformatics website. [file 1471-2105-15-141-S2.zip › 1394847754114233_MOESM2_ESM/A431_MCF7/figures/22944.pdf]

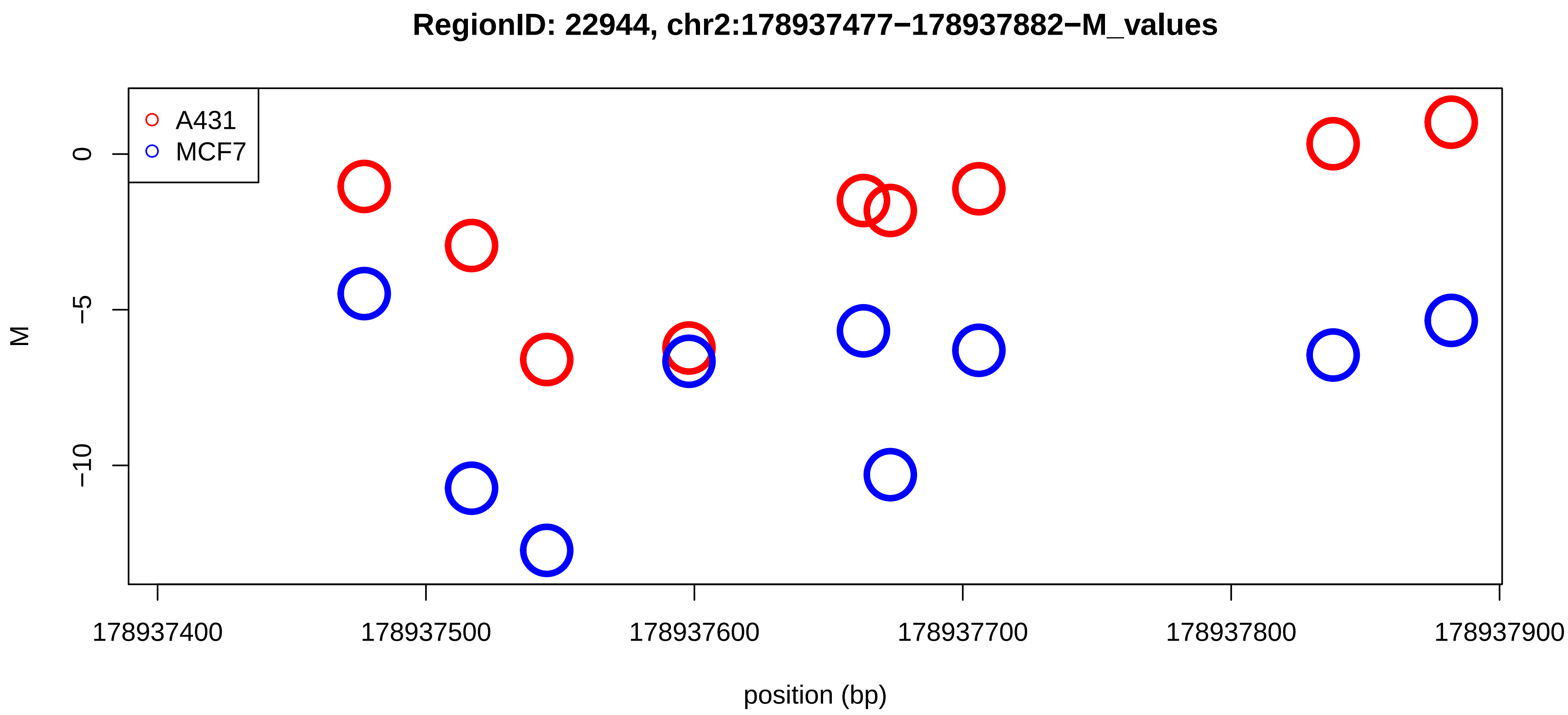

RegionID: 22944, chr2:178937477−178937882−M_values
A431
MCF7
M
0
−5
−10
178937400
178937500
178937600
178937700
178937800
178937900
position (bp)

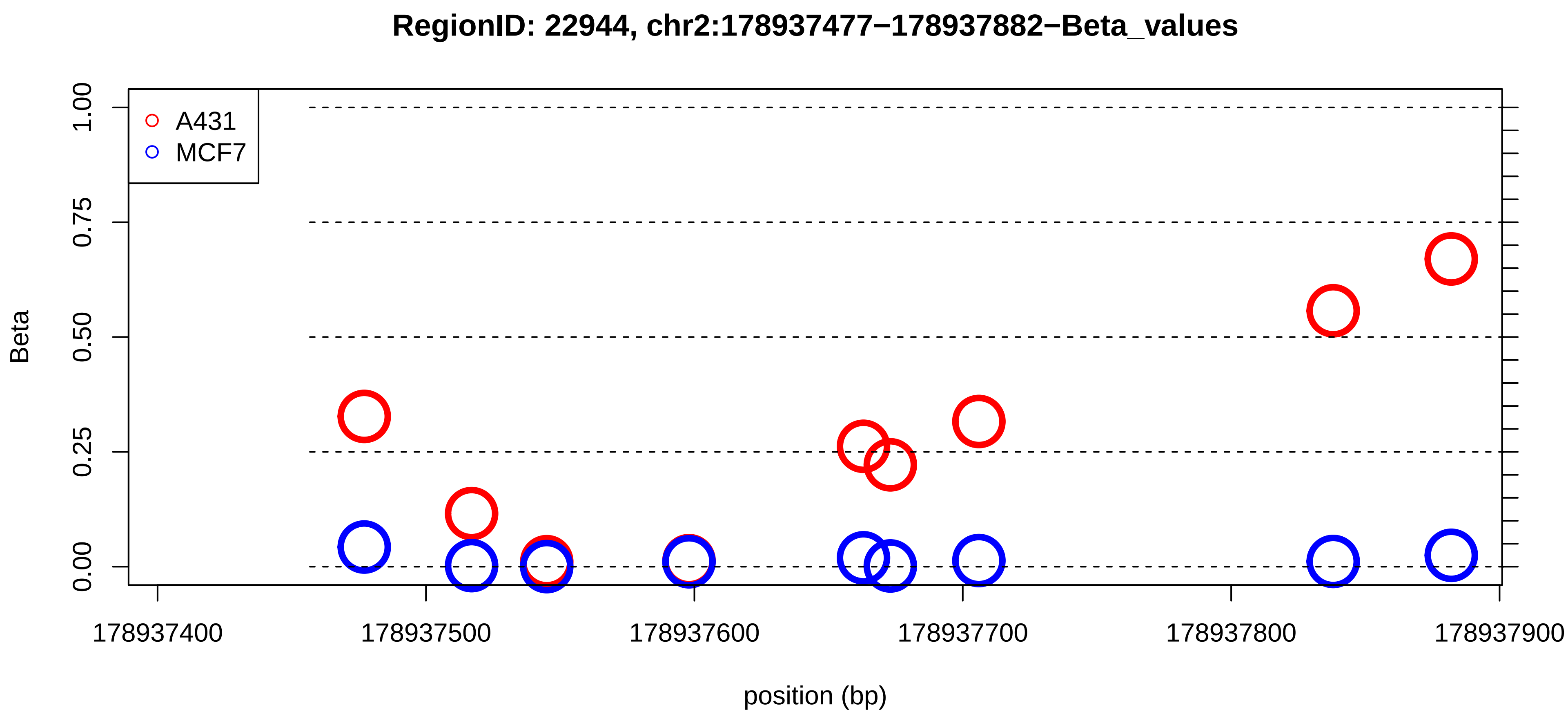

RegionID: 22944, chr2:178937477−178937882−Beta_values
A431
MCF7
Beta
0.00
0.25
0.50
0.75
1.00
178937400
178937500
178937600
178937700
178937800
178937900
position (bp)

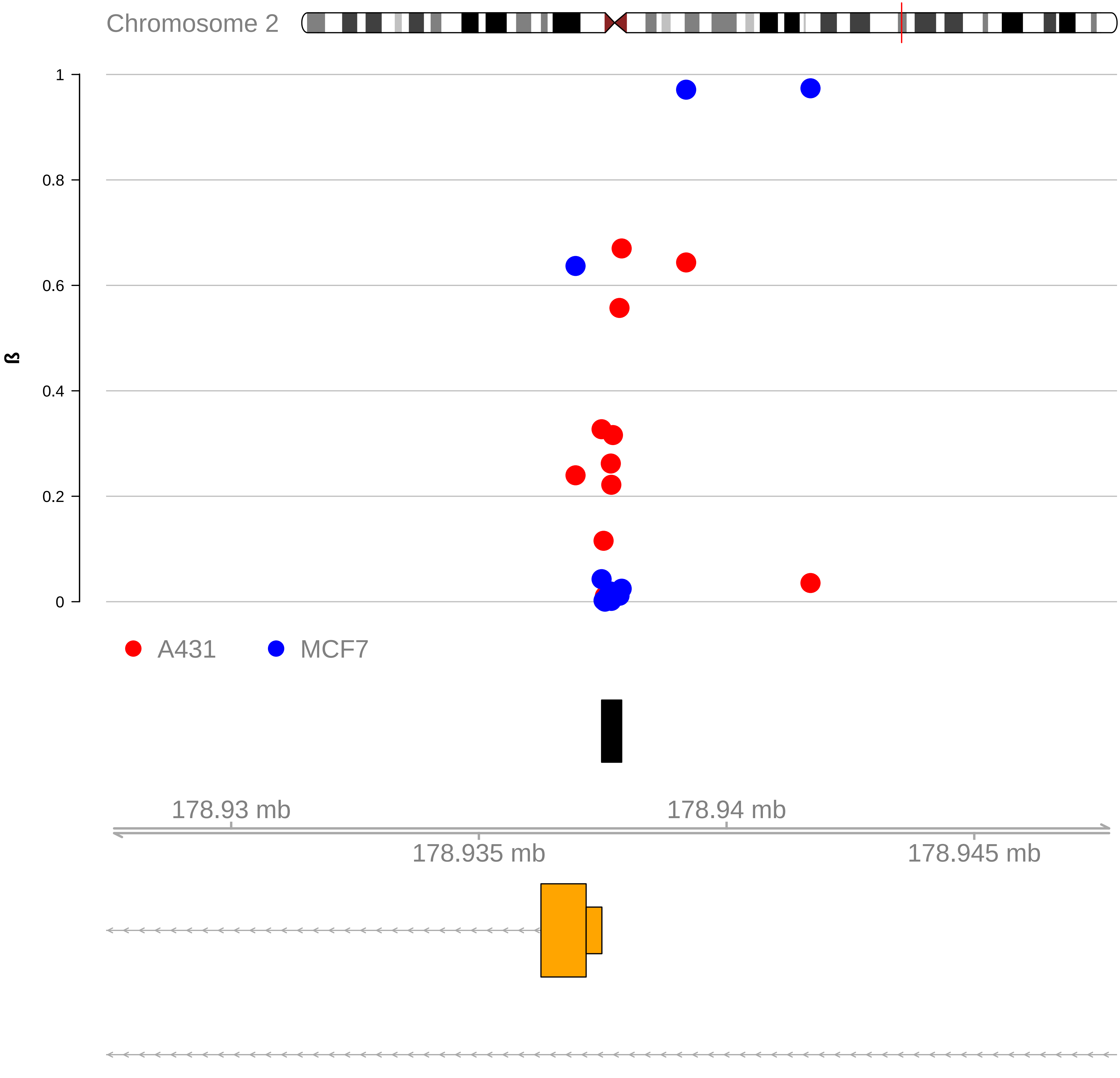

Chromosome 2
1
0.8
0.6
0.4
0.2
0
ß
A431
MCF7
178.93 mb
178.94 mb
178.935 mb
178.945 mb
